# Nanophthalmos patient with a THR518MET mutation in *MYRF*, a case report

**DOI:** 10.1186/s12886-020-01659-8

**Published:** 2020-10-01

**Authors:** Joshua Hagedorn, Armin Avdic, Michael J. Schnieders, Benjamin R. Roos, Young H. Kwon, Arlene V. Drack, Erin A. Boese, John H. Fingert

**Affiliations:** 1 Carver College of Medicine, University of Iowa, Iowa City, IA USA; 2 Department of Biochemistry, Carver College of Medicine, University of Iowa, Iowa City, IA USA; 3 Department of Ophthalmology and Visual Sciences, Carver College of Medicine, University of Iowa, Iowa City, IA USA; 4 Institute for Vision Research, University of Iowa, Iowa City, IA USA; 5 Medical Education and Research Facility, University of Iowa, 375 Newton Road, Iowa City, IA 52242 USA

**Keywords:** Nanophthalmos, Myelin regulatory factor, *MYRF*, Case report

## Abstract

**Background:**

Nanophthalmos has a significant genetic background and disease-causing mutations have been recently been reported in the myelin regulatory factor (*MYRF*) gene. We report clinical features in a patient with nanophthalmos and a Thr518Met *MYRF* mutation.

**Case presentation:**

A three-year-old male was discovered to have nanophthalmos after first presenting to the emergency department for a frontal headache, eye pain, emesis, and lethargy. Imaging studies (CT and MRI) were negative except for increased posterior fossa cerebrospinal fluid. Subsequent examinations revealed nanophthalmos (short axial eye lengths 18.1 mm OD and 18.3 mm OS), microcornea, and a large crystalline lens. Peripheral chorioretinal pigment abnormalities were also observed. He experienced episodes of marked ocular hypertension (53 mmHg OD and 60 mmHg) likely due to intermittent angle closure precipitated by nanophthalmos. The ocular hypertension was responsive to topical medicines. Genetic analysis of known nanophthalmos genes *MFRP* and *TMEM98* were negative, while a novel mutation, Thr518Met was detected in *MYRF*. The Thr518Met mutation was absent from 362 matched normal controls and was extremely rare in a large population database, allele frequency of 0.000024. The Thr518Met mutation altered a highly conserved amino acid in the MYRF protein and three of four algorithms suggested that this mutation is likely pathogenic. Finally, molecular modeling showed that the Thr518Met mutation is damaging to MYRF structure. Together these data suggest that the Thr518Met mutation causes nanophthalmos.

**Conclusions:**

Nanophthalmos may present at an early age with features of angle closure glaucoma and a Thr518Met mutation in *MYRF* was detected in a patient with nanophthalmos. Prevalence data, homology data, mutation analysis data, and protein modeling data suggest that this variant is pathogenic and may expand the phenotypic range of syndromic nanophthalmos caused by *MYRF* mutations to include central nervous system abnormalities (increased posterior fossa cerebrospinal fluid).

## Background

The term nanophthalmos is derived from the Greek word “nano” meaning “dwarf,” and is defined by high hyperopia (> + 5 diopters) and/or short axial eye length (< 20 mm). Nanophthalmos has a strong genetic basis. Genes for autosomal recessive (*MFRP* and *PRSS56*) and autosomal dominant (*TMEM98*) nanophthalmos have been discovered [1]. Recently, variants in the myelin regulatory factor (*MYRF*) gene were identified as another cause of autosomal dominant nanophthalmos and may be responsible for 2–18% of cases of disease [2–5]. The *MYRF* gene encodes a membrane protein that is subject to autocleavage, releasing a transcription factor protein fragment that stimulates expression of genes important for myelination of CNS nerve fibers. [6] Variants in *MYRF* had been previously associated with cardiac defects (scimitar syndrome, dextrocardia), [7–9] urogenital malformations [7–9], diaphragmatic hernia [9], and encephalopathy. [10] In 2019, 10 unique *MYRF* variants were detected in nanophthalmos patients, including 9 that cause premature termination of translation via frameshifts due to insertions, deletions, splicing defects, or nonsense variants [2, 3, 5]. Although many of these nanophthalmos patients had dextrocardia and urogenital malformations [2, 4], some had isolated nanophalmos. [3] De novo *MYRF* variants have also been detected in three nanophthalmos patients that are the offspring of normal parents [5], suggesting that de novo variants may be a relatively common feature of nanophthalmos caused by the *MYRF* gene. Here we report a patient with nanophthalmos and a novel mutation in the recently discovered *MYRF* gene.

## Case presentation

### Clinical examination

A 3-year-old male (Nan3) presented to the emergency department with a frontal headache, eye pain, emesis, and lethargy. Physical exam was essentially normal with no focal neurologic deficits. Work up included a CT scan and MRI, which identified increased posterior fossa fluid. A lumbar puncture was also performed with normal opening pressure. Eye examination was limited due to patient agitation, but did not reveal any abnormal findings; his visual acuity was at least fix and follow OU, and the dilated eye exam was only notable for peripheral geographic chorioretinal scars OU. Toxoplasmosis serology was obtained, which was negative, and follow-up was arranged with ophthalmology. Both neurology and neurosurgery were following on an as needed basis.

**Fig. 1:**
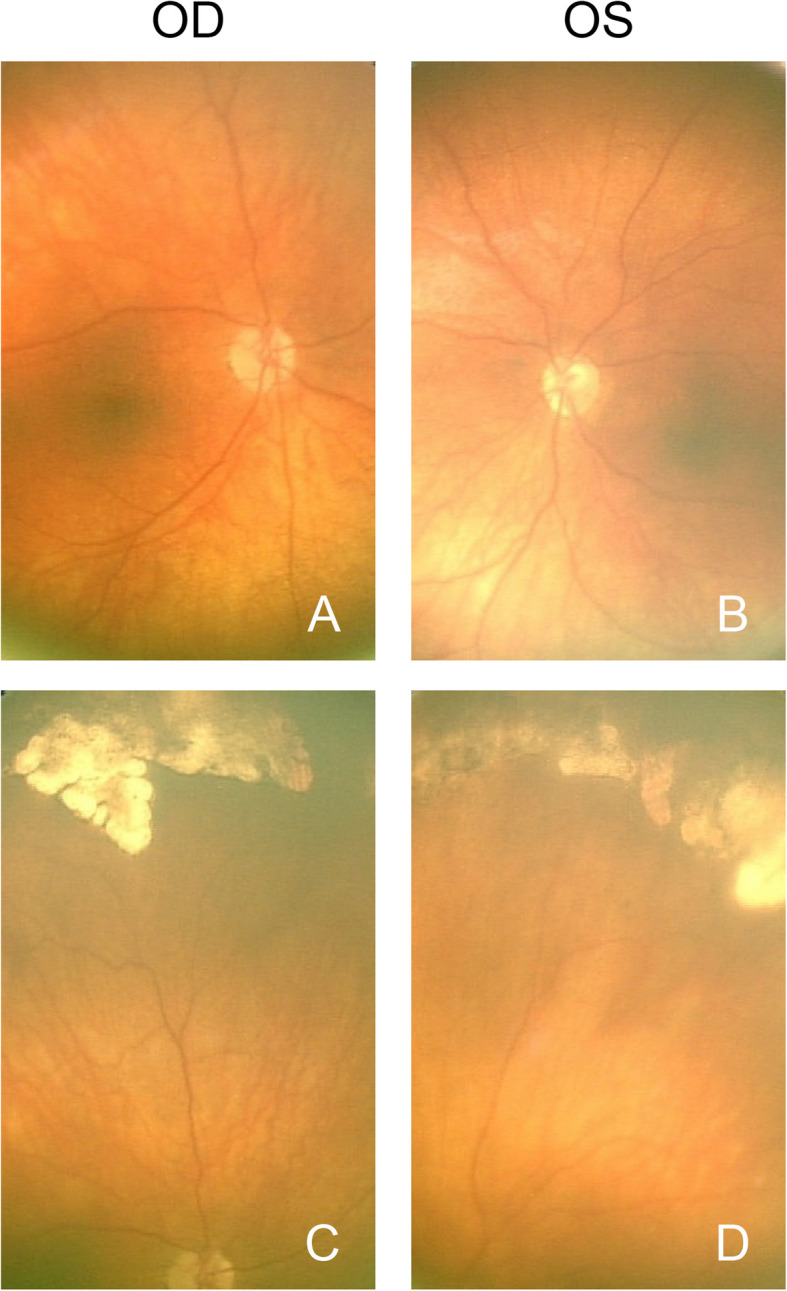
Retinal photographs of patient (Nan3) with Thr518Met variant in *MYRF*. RetCam photographs of the posterior pole OD (**a**) and OS (**b**) and of the peripheral retina OD (**c**) and OS (**d**)

**Fig. 2:**
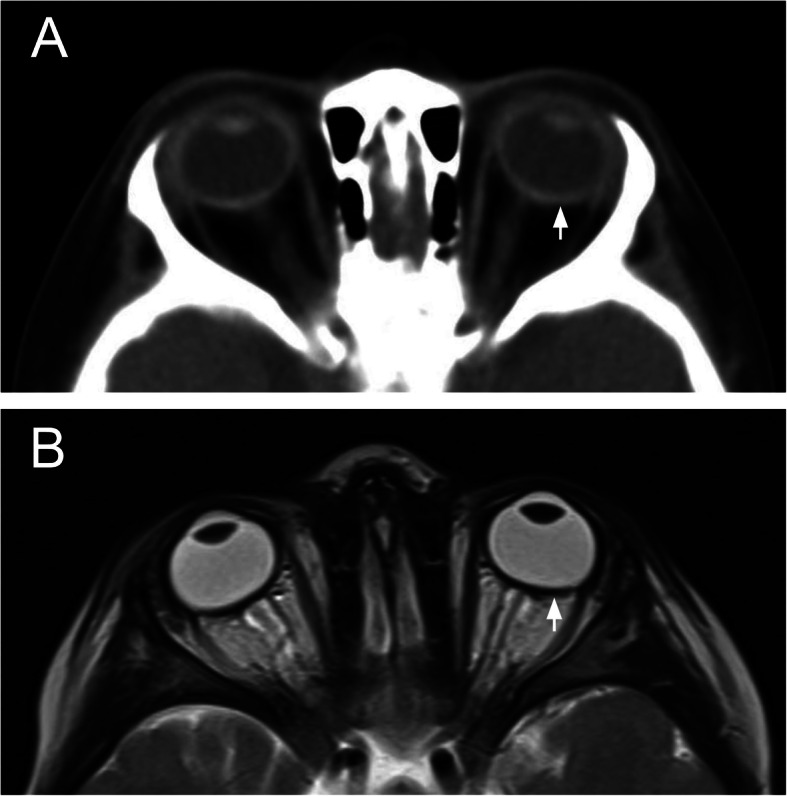
CT and MR imaging of patient (Nan3) with Thr518Met variant in *MYRF*. Axial CT (**a**) and axial T2 MRI (**b**) scans demonstrate short axial eye length and scleral appearance consistent with ultrasound measures and a diagnosis of nanophthalmos. A white arrow indicates sclera which is hyperdense in the CT image (**a**) and hypointense in the MR image (**b**)

At follow up eye examination, Nan3’s symptoms had resolved. His visual acuities were 2.4cy/cm (20/260) OD and 6.5cy/cm (20/94) OS by Teller cards, and intraocular pressure (IOP) was 7 mmHg OD and 8 mmHg OS by iCare tonometer. Cycloplegic refraction revealed high hyperopia (+ 7.00 + 0.50 × 090 OD and +  7.00 + 0.50 × 090 OS). On subsequent exam under anesthesia, his pachymetry readings were thick at 640 μm OD and 616 μm OS, and the horizontal white-to-white measures were small at 10.5 mm OD and 10.6 mm OS. Gonioscopy showed angles open to the scleral spur OD and with 2 clock-hours of angle closure OS. The anterior segment examination was otherwise normal. The optic nerves OU had mild pallor and cup-to-disc ratios of 0.4 OD and 0.3 OS respectively (Fig. 1a and b). As previously noted, there were chorioretinal scars in the periphery of both eyes (Fig. 1c and d). A-scan ultrasound measured short axial eye lengths of 18.1 mm OD and 18.3 mm OS), a thick sclera (2.0 mm OU), and a relatively large lens (4.3 mm OD and 4.1 mm OS). CT and MRI scans also demonstrate short axial eye lengths and thick sclera (Fig. 2). He did not show any signs of primary congenital glaucoma, including buphthalmos, Haab’s striae, or corneal edema and was diagnosed with nanophthalmos based on axial eye length measurements.

At subsequent follow-up appointments during the next year, Nan3 had several episodes of unilateral high IOPs (once in the right eye to 53 mmHg, three times in the left ranging from 42 to 60 mmHg). Topical medications (timolol-dorzolamide and latanoprost) were successful in lowering intraocular pressure during 9 months of follow-up. Based on his clinical course and measurements, Nan3 was diagnosed with nanophthalmos, microcornea, and likely intermittent angle-closure glaucoma. Close follow-up was arranged to monitor his IOP and iridocorneal angles and the need for surgical intervention. Unfortunately, he was lost to follow-up.

Nan3 has no known family history of nanophthalmos. His parents were unavailable for examination, but are not known to be highly hyperopic or have a history of angle closure glaucoma.

### Genetic testing

We tested Nan3’s DNA for coding sequence variants in *TMEM98* and *MYRF* using standard Sanger sequencing, and *MFRP* was also tested by a commercial laboratory (Bayler Miraca, Houston, TX). No variants were detected in *TMEM98* or *MFRP*, however, we detected two heterozygous variants in *MYRF*, a synonymous change (rs149803 - Pro263Pro) and a missense change (Thr518Met - rs562652459).

### Variant analysis

**Fig. 3:**
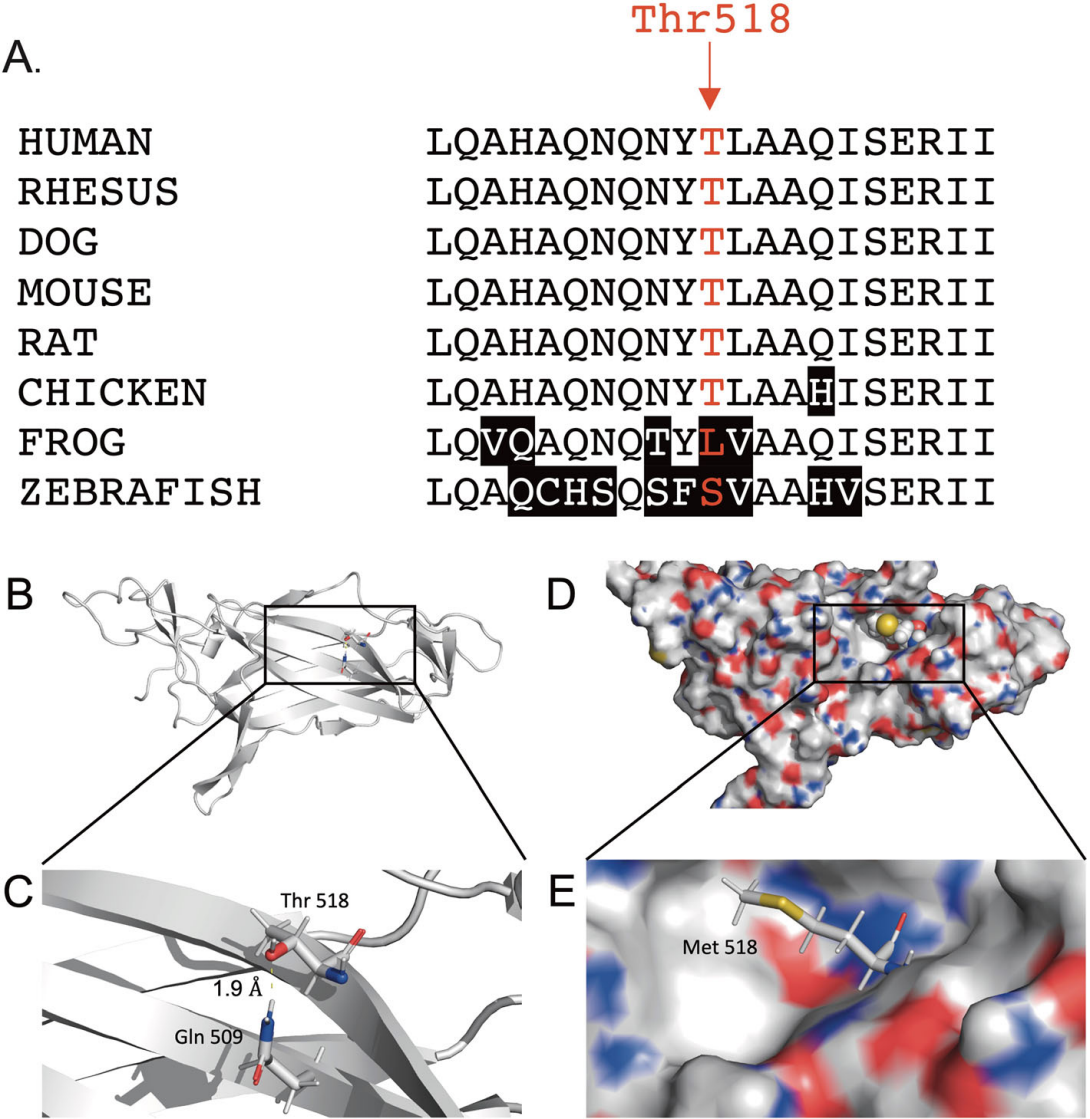
Analysis of *MYRF* variant, Thr518Met. **a**. Homology of MYRF sequences. Comparison of MYRF protein sequence from several organisms demonstrates that the threonine amino at position 518 is highly conserved across many species. Position 518 is indicated by red letters. Divergent amino acids are indicated by black background. **b-e**. Structural modeling of the Thr518Met *MYRF* variant. Beginning from an MYRF crystal structure, variant of residue 518 from Thr to Met followed by side-chain repacking demonstrates the loss of a canonical hydrogen-bond between Thr518 and Gln509 (1.9 Å) that stabilizes the interaction between two *β*-sheets within the *MYRF* DNA-binding domain (**b** and **c**). This suggests that a functional consequence of this variant is diminished folding stability, which may alter DNA-binding affinity and result in a reduction in transcriptional control. The mutant structure with a Met residue at position 518 **(d** and **e**) is surface exposed and can be accommodated without a substantial change in conformation

We evaluated the potential pathogenicity of the Thr518Met variant in the *MYRF* gene by assessing its prevalence in control subjects with normal eyes. No instances of the Thr518Met variant were detected in the exomes of 362 normal control subjects from the University of Iowa. Moreover, the Thr518Met variant was only rarely observed in the gnomAD database at an allele frequency of 0.000024 in a non-Finnish European population (*n* = 61,642, gnomad.broadinstitute.org) as would be expected for a variant that causes nanophthalmos. Parental samples were not available for segregation studies or to determine if Thr518Met was a de novo variant in the *MYRF* gene. Nan3’s parents reported no history of high hyperopia or angle closure glaucoma, which would be consistent with a de novo *MYRF* variant in Nan3. We further explored the pathogenicity of Thr518Met with a homology analysis. The Thr518Met variant alters an amino acid in a DNA-binding domain of MYRF that has been conserved across a diverse range of mammals and vertebrate animals (Fig. 3a). We also investigated the pathogenesis of Thr518Met with variant analysis algorithms. Three of four algorithms suggested that the Thr518Met variant is deleterious (Table 1). Finally, we modeled the tertiary structure of the MYRF protein with and without the Thr518Met variant using its solved crystal structure (PDB ID: 5YHU) [11] and refinement algorithms based on the polarizable AMOEBA force field as we have previously described [12]. Our analysis showed that the Thr518Met variant replaces a polar amino acid (threonine) with a larger, hydrophobic amino acid (methionine), which results in the loss of a hydrogen bond between two MYRF DNA-binding domain *β*-strands (Fig. 3b). Together these data suggest that the Thr518Met variant in *MYRF* may cause nanophthalmos by destabilizing the fold of its DNA-binding domain and altering its function as a transcription factor.

**Table 1:** Features of nanophthalmos patient (Nan3) with a Thr518Met *MYRF* variant. Allele frequencies of *MYRF* variants Pro263Pro and Thr518Met were obtained from gnomad.broadinstitute.org

**Ocular features relevant to nanophthalmos**	**OD**	**OS**
Hyperopia	+ 7.00 + 0.50 × 90	+ 7.00 + 0.50 × 90
Central corneal thickness	640 μm	616 μm
Ultrasound measurements		
Axial eye length	18.1 mm	18.3 mm
Anterior chamber depth	2.2 mm	2.4 mm
Lens	4.3 mm	4.1 mm
Scleral thickness	1.95 mm	1.95 mm
Ocular hypertension		
Maximum IOP	53 mmHg	60 mmHg
**Other ocular features**		
Cornea		
Horizontal diameter	10.5 mm	10.5 mm
Retina	Chorioretinal scars	
**Systemic features**		
Central Nervous System	Posterior fossa cyst	
**Genetic testing results**		
*MYRF*: c.1553C > T, p.Thr518Met (rs562652459)		
Allele frequency		
University of Iowa database (*n* = 362 controls)	0	
gnomAD database (*n* = 61,642 Non-Finnish Europeans)	0.000024	
Variant analysis (estimation of pathogenicity)		
Blosum62	−1	
Polyphen	0.677 (Probably damaging)	
CADD	− 21.5 (1% most deleterious)	
SIFT	Tolerated	

## Discussion and conclusions

Syndromic cases of nanophthalmos due to *MYRF* variants have included cardiac and genitourinary abnormalities [2, 4]. Here we expand the phenotypic range of *MYRF* variants by reporting a patient with nanophthalmos along with retina abnormalities and a posterior fossa cyst.

Nan3 meets diagnostic criteria for nanophthalmos. It is likely that this patient’s presenting symptoms of headache, eye pain, emesis, and lethargy were caused by intermittent angle-closure predisposed by nanophthalmos. Nan3 also had microcornea and a relatively large lens, both exacerbating the anterior segment crowding of nanophthalmos and further increasing risk for angle closure. Hyperpigmented chorioretinal scars in the periphery of the retina resembling cobblestone degeneration, as seen in this patient, may be caused by lobular choroidal infarcts during episodes of high IOP. However, gonioscopy during episodes of high IOP did not show iris bombé or occluded angles, and there was no evidence of phacodonesis that might promote intermittent angle closure. The specific cause of elevated IOP in this patient remains unclear and in addition to angle closure, other diagnoses such as Posner-Schlossman or juvenile open angle glaucoma might be included on a differential diagnosis. The markedly early-onset of symptoms in this patient is also unusual and may be a feature of *MYRF*-related disease.

Nan3 is the only nanophthalmos patient reported to have a Thr518Met variant in *MYRF* and no segregation data is available to provide support for its pathogenicity. It remains possible that the Thr518Met is a rare, benign variation that was incidentally identified in Nan3. However, the sum of the data (prevalence data, homology data, mutation analysis data, protein modeling data) suggests that this variant may cause syndromic nanophthalmos. Although additional functional and/or animal studies of this variant will be ultimately be needed to prove its pathogenicity, our report of the Thr518Met variant provides additional evidence to support the recent association between *MYRF* and nanophthalmos.

## Data Availability

The datasets used and/or analysed during the current study are available from the corresponding author on reasonable request.

## Abbreviations

THR: Threonine
MET: Methionine
*MYRF*: Myelin regulatory factor gene
*MFRP*: Membrane frizzled-related protein gene
*TMEM98*: Transmembrane 98 protein gene
*PRSS56*: Serine protease 56 gene
PRO: Proline
OD: Right eye
OS: Left eye
OU: Both eyes
IOP: Intraocular pressure
